# Interplay between volemic balance and the intestinal tract: insights on biomarkers and diagnostic tests used to assess intestinal morphofunctional barrier

**DOI:** 10.1590/1414-431X2025e15041

**Published:** 2026-01-30

**Authors:** V.L.M. Neto, I.C.D. Araújo, T.B.M. Rôla, P.J.C. Magalhães, F.A.P. Rodrigues, A.A.M. Lima, A.A. Santos

**Affiliations:** 1 Programa de Pós-graduação em Ciências Médicas, Faculdade de Medicina, Universidade Federal do Ceará, Fortaleza, CE, Brasil; 2 Programa de Pós-graduação em Ciências Cardiovasculares, Faculdade de Medicina, Universidade Federal do Ceará, Fortaleza, CE, Brasil; 3 Instituto Federal de Educação, Ciência e Tecnologia do Ceará, Fortaleza, CE, Brasil; 4 Núcleo de Biomedicina, Universidade Federal do Ceará, Fortaleza, CE, Brasil

**Keywords:** Body fluid volume regulation, Biomarkers, Intestinal morphofunctional barrier, Lactulose/mannitol, Mass spectrometry

## Abstract

Volemic control is essential for maintaining tissue perfusion and fluid homeostasis, with cardiorenal and endothelial mediators regulating intravascular composition, often impaired in pathological states. Notably, intestinal epithelial cells are highly sensitive to volume fluctuations, resulting in changes in intestinal permeability that may not be detected by current diagnostic methods. This review offers a comprehensive description of the main mediators involved in volemic regulation, their impact on intestinal morphofunctionality, and specific details regarding epithelial cells. Additionally, key biomarkers - especially lactulose/mannitol - for assessing intestinal barrier disruption are highlighted, and a novel approach is proposed using liquid chromatography-mass spectrometry to investigate gut alterations in heart failure and exercise-induced stress, which are silent and neglected conditions with significant repercussions on intestinal barrier function.

## Introduction

The cardiorenal axis, modulated by neural, hormonal, and humoral mediators, regulates blood volume status by mediating the transport and distribution of essential substances in the body and the excretion of metabolic waste products (1). The cardiovascular system regulates tissue perfusion, while the kidneys play an essential role in maintaining the volume and composition of body fluids by targeting osmolality, electrolyte balance, and acid-base homeostasis in the extracellular milieu (2). Such actions operate bidirectionally under both physiological and pathophysiological conditions, mediated by sympathetic activity (catecholamines, e.g., noradrenaline and adrenaline), the renin-angiotensin-aldosterone system (e.g., angiotensin II), endothelin, natriuretic peptide (NP) family [well-known atrial natriuretic peptide (ANP), B-type natriuretic peptide (BNP), and C-type natriuretic peptide (CNP)], and enteric peptides (guanylin and uroguanylin), although the underlying mechanisms, receptors, and pleiotropic effects are not yet fully understood (1,3).

Subtle changes in fluid volume or composition can affect cardiorenal function. In this context, fluid and solute intake also plays a critical role, particularly highlighting the complex morphofunctional structure of the intestinal epithelium, which comprises the machinery responsible for the absorption of substances and fluids from the intestinal lumen. Conversely, impaired intravascular distribution negatively impacts the function of intestinal cells (4). This review aims to underscore the presence of a homeostatic network of integrated physiological interactions within the cardiovascular-renal-intestinal axis, wherein body fluid composition serves as a bidirectional modulating sensor.

Investigating the pathophysiology of the intestinal epithelium is complex due to its dynamic, self-renewing structure, which precisely regulates nutrient and electrolyte absorption while serving as a selective barrier with immunomodulatory functions (5,6). Disturbances in molecular mechanisms orchestrated by junctional proteins, such as claudins, occludins, and zonula occludens (ZO), alter the paracellular permeability, leading to the loss of intestinal barrier function, as demonstrated in both clinical and preclinical studies (7). This contributes to increased intestinal permeability, observed in several disease processes, but with limited diagnosis (8). Reliable methods are needed to assess the physiological changes occurring within intestinal morphofunctional tight junctions (TJs). Clinical and preclinical studies have been making progress in the validation of intestinal biomarkers for assessing epithelial dysfunction, notably the lactulose/mannitol excretion ratio (L:M), particularly in the context of intestinal permeability (9). However, while this approach is widely applied in inflammatory bowel diseases (10), it is rarely used in studies investigating the gastrointestinal symptoms associated with impaired flow, distribution, and perfusion induced by heart failure and/or physiological stress from physical exercise.

This review aims to delineate cardiorenal markers and their role in volemic control and discuss the implications of intravascular volume variations on intestinal barrier dynamics. The application of analytical methods to evaluate intestinal permeability in heart failure and physical exercise are also discussed, the former being a physiopathological condition and the latter a physiological stressor, both of which are still underexplored in the literature, particularly regarding alterations in intravascular content.

## Volemia regulation

### Volemic distribution in body fluid compartments

It is estimated that water accounts for approximately 50 to 70% of the adult human body, distributed across two main compartments: the intracellular compartment, which is the largest one, and the extracellular compartment. The intracellular compartment refers to the fluid within cells, while the extracellular fluid (ECF) includes the plasma, i.e. the colorless fluid part of blood without cellular components, the interstitial fluid (fluids that flow within the microscopic spaces between cells), and other fluids such as the lymph, saliva, ocular fluid, secretions from glands and the digestive system, cerebrospinal fluid, and the fluids excreted by the skin and kidneys (11).

**Figure 1:**
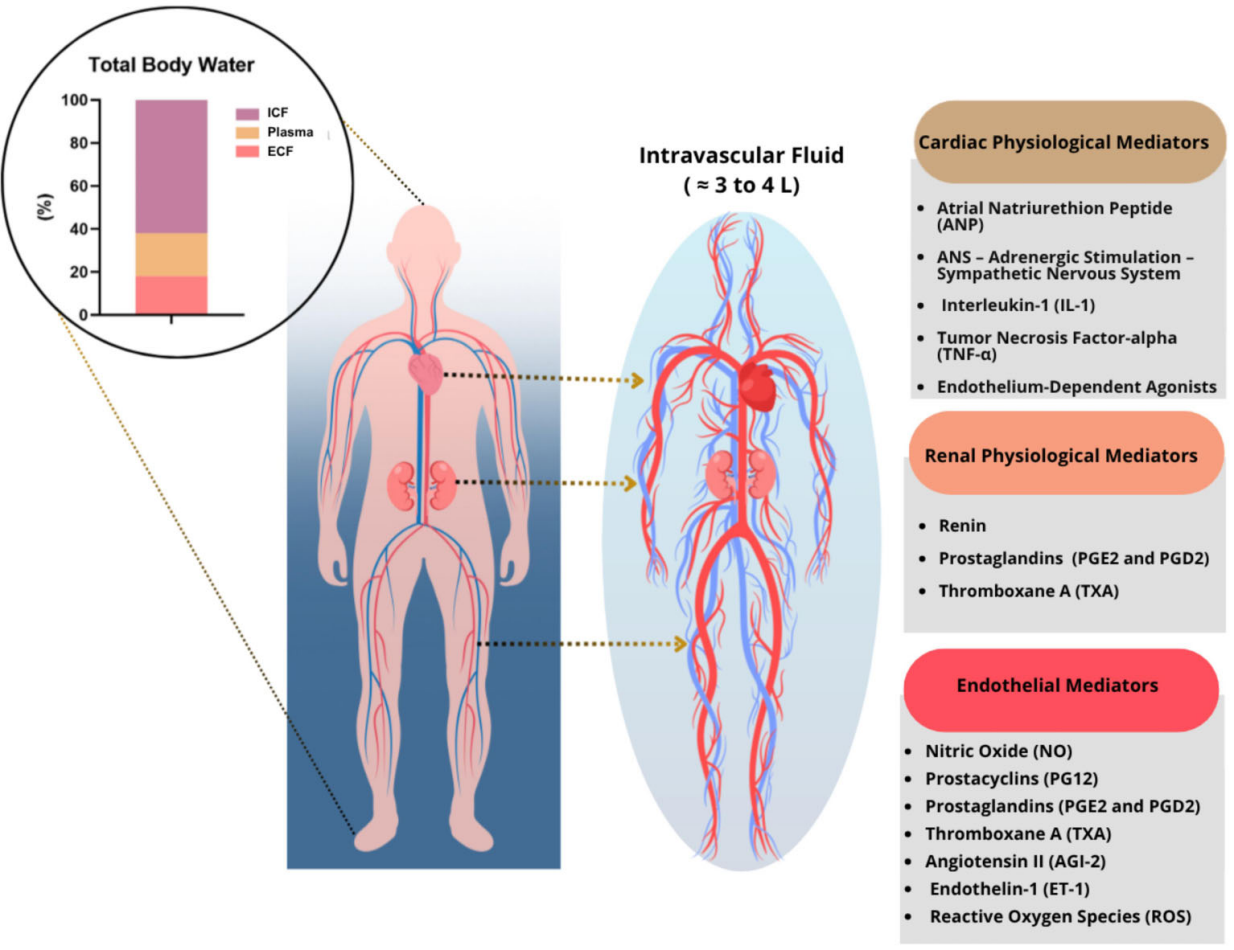
Compartmentalization of body volumes and the respective modulators that control composition and flow. The influence of input and output of the cardiovascular circuit is shown, as well as the list of mediators that control the composition and flow based on their influence on the volemic system. ICF: intracellular fluid; ECF: extracellular fluid.

The physiological control of fluid volume is mostly exerted on the intravascular compartment, i.e., the plasma, while the fluid within the interstitial space, i.e., the interstitial fluid, represents the major physiological target in the extravascular compartment. Blood plasma volume (2.5 to 3 L) accounts for almost 20% of the total extracellular fluid. As for total body water, 62% on average is intracellular water (26 L of the body's 42 L for an 80-kg man) and 38% is of extracellular origin (11,12) (Figure 1).

The regulation of the intravascular volume is mediated by the interaction between afferent and efferent mechanisms that target solute and water balance in the extracellular compartment, with sodium acting as the main solute determining the extracellular osmolarity (13). Since the kidneys are primarily responsible for the urinary excretion of sodium and water, they play a central role in maintaining the intravascular volume. Under normal homeostatic conditions, the heart and kidneys interact through humoral atrio-renal pathways to promote increased urinary sodium and water excretion (14).

Changes in plasma volume occur in several clinical situations, including physiological conditions, such as healthy individuals who practice physical activity and pregnancy, and in pathological conditions, such as chronic anemia, liver cirrhosis, and heart failure (12,15).

### Cardiorenal regulation of body volemia

The cardiorenal axis plays a central role in regulating the volume and composition of body fluids. The primary function of the cardiovascular system is to ensure adequate blood flow and distribution of essential substances to tissues according to their physiological demands. Additionally, it contributes to homeostatic regulation by maintaining fluid balance and modulating the supply of oxygen and nutrients under various physiological conditions, thereby ensuring proper tissue perfusion (16).

The renal system plays a vital role in regulating both the volume and composition of body fluids. In their role of maintaining homeostasis through the regulation of extracellular osmolality, electrolyte concentrations, and acid-base balance, the kidneys are highly sensitive to deviations in these parameters, that is, they respond appropriately to diverse physiological stimuli in a way that supports survival (17).

One of the renal effector mechanisms for maintaining body fluid homeostasis involves the control of the glomerular filtration rate (GFR). Signaling mechanisms contribute to balance the excretion of sodium ions (Na^+^) and water by the kidneys, such as prostaglandins, endothelium-derived factors, the classic renin-angiotensin-aldosterone system (RAAS), and ANP (18).

In a clinical environment, there is a broad consensus on the need for detailed monitoring of blood volume changes, with particular attention to key cardiorenal variables. Baseline renal function serves both as a predictor of response to diuretic therapy and as a marker of poor prognosis. Simultaneous variations in renal function, extracellular sodium levels, congestion status, and their interdependence are fundamental parameters, analyzed in conjunction with volemia, for assessing cardiorenal balance (19). Supplementary Table S1 presents a summary of the main mediators that influence body volume status; these markers respond to intrinsic factors (solute concentration) and/or blood pressure and humoral index, being released according to physiological needs, especially to maintain intra- and extravascular homeostasis (e.g., intravascular volume).

### Natriuretic peptides and volume homeostasis

The natriuretic peptide system functions as an important endocrine, autocrine, and paracrine system, acting as a natural antagonist of the renin-angiotensin-aldosterone system and contributing to the maintenance of cardiovascular homeostasis. It primarily consists of three genetically distinct but structurally related peptides: atrial ANP, B-type natriuretic peptide (BNP), and C-type natriuretic peptide (CNP) (20). ANP is predominantly synthesized in the atria and released in response to atrial distension. BNP is synthesized and secreted mainly by ventricular myocytes in response to volume and pressure overload of these chambers after volume overload, leading to stretching of the ventricular wall (21). CNP is secreted primarily by the vascular endothelium upon stimulation by proinflammatory cytokines (e.g., interleukin-1 and tumor necrosis factor) and endothelium-dependent agonists (e.g., acetylcholine) (22).

NPs play a fundamental role in maintaining blood pressure (BP) and intravascular volume. BP control can be achieved through the regulation of vascular tone, caused by a direct relaxing effect of NPs on vascular smooth muscle cells (23). Additionally, NPs help regulate BP by suppressing the RAAS, reducing sympathetic tone, and inhibiting endothelin-1 (ET-1) secretion (20). In this context, NPs play a fundamental role in regulating intravascular volume, influencing renal electrolyte and water balance, and directly mediating vascular endothelial permeability (24). Through its vasodilator properties, ANP increases the glomerular filtration rate, while reducing sodium reabsorption in the collecting tubule by acting as an antagonist of the RAAS (25).

The natriuretic peptide system has gained increasing prominence in hospital care, particularly for detection of cardiac dysfunction. Plasma measurement of BNP and its N-terminal fragment (NT-proBNP) - both active hormones released by the cardiac ventricles in response to volume and pressure overload - has shown strong correlation with heart failure severity. Elevated levels of these biomarkers are useful for diagnosis, monitoring treatment response, and assessing prognosis (18,22) (Figure 1).

The interaction between ANP and oxytocin has been recently investigated in the context of right atrial stretch on the gastric emptying of liquid. According to Palheta et al. (25), the stretching of a balloon in the right atrium releases both ANP and oxytocin, significantly delaying the gastric emptying of liquid. In their preclinical model, the right atrial stretch was experimentally induced, and specific antagonists were administered to block the effects of these hormones. Inhibiting their actions attenuated the gastric emptying delay, indicating that ANP and oxytocin have complementary roles in modulating the gastrointestinal motility (26). According to Aikins et al. (27), oxytocin has cardiovascular effects beyond its classical roles and is also synthesized in the heart and major vessels, where it influences vascular tone and induces negative chronotropic effects via ANP release. This interaction suggests that oxytocin may modulate hydroelectrolytic homeostasis and gastrointestinal functions through paracrine and endocrine mechanisms. Supplementary Table S2 summarizes the main neurohormonal, humoral-mediated, and local signaling mediators that modulate volume status.

### Gut-derived natriuretic peptides and their influence on volume status

The network of agents involved in blood volume regulation is complex. As an example, enteric hormones known as intestinal natriuretic factors have also been identified. In early studies, the biological properties of guanylin and uroguanylin were identified and believed to be potential intestinal natriuretic factors. It was observed that the ingestion and absorption of NaCl triggered the release of a substance from the intestine that acted on the kidneys, functioning as a regulatory mechanism for sodium excretion in the postprandial state (28).

The existence of an endocrine axis linking the intestine to the kidney was first suggested in the early 1980s, based on studies using a semi-purified extract of heat-stable *Escherichia coli* toxin. In addition to its well-known intestinal effects, the extract also produced renal effects such as diuresis, natriuresis, and kaliuresis. These findings were later confirmed through experiments using the purified form of the toxin (29).

Peptides of the guanylin family - particularly uroguanylin, secreted by intestinal enterochromaffin cells - function as hormones within an endocrine axis linking the gastrointestinal tract to the kidney (the entero-renal axis), as well as within an intrarenal paracrine system (30). Lima and Fonteles reported that the activation of guanylate cyclase C (GC-C) receptors by these peptides triggers postprandial sodium excretion (as previously documented), establishing a crucial link between two vital systems responsible for maintaining homeostasis in the face of external environmental changes (29). This highly integrated mechanism further enables small concentrations of atrial ANP to act synergistically with low concentrations of guanylin or uroguanylin - which alone do not induce natriuresis - to promote significant natriuretic effects (31).

Sodium balance is essential for the regulation of intravascular volume. Therefore, the guanylin family may play a role in the pathogenesis of hypertension and heart failure. In pathophysiological conditions involving salt and water retention, such as heart failure, plasma levels of uroguanylin are elevated (32). This is likely a compensatory response, resulting from enhanced secretion of uroguanylin by other organs, such as the heart and gastrointestinal tract, to promote diuretic and natriuretic effects (29,30). Thus, it is presumed that the physiological functions of these peptide hormones are critically involved in the regulation of sodium homeostasis, particularly when animals are exposed to excess sodium chloride levels from the environment or diet, making them an active and integral component of the complex fluid balance regulatory system.

### Neuroendocrine influence on body fluids

The hypothalamus regulates ECF osmolality and volume through osmo- and baroreceptors. When plasma osmolality increases or baroreceptor signaling decreases, neural and hormonal pathways trigger thirst and ADH secretion to reduce water loss via the kidneys. Conversely, when ECF osmolality decreases or volume expands, these mechanisms suppress thirst and ADH release, promoting water excretion to restore balance (13).

The endocrine system, in turn, secretes hormones which, via the renin-angiotensin-aldosterone system, become a critical regulator of blood volume, electrolyte balance, and systemic vascular resistance, being responsible for acute and chronic changes in the intravascular volume. This system comprises three important compounds: renin, angiotensin II, and aldosterone, the levels of which increase in plasma in response to a decreased renal blood perfusion pressure in the renal arteries, subsequently raising blood pressure. In contrast, higher perfusion pressure values and increased sodium supply to the distal convoluted tubule inhibit these systemic signaling processes (25) (Supplementary Table S1).

Renin is released into the circulation in response to several physiological stimuli. These include: changes in renal perfusion pressure detected by mechanoreceptors in the afferent arteriolar wall; increased sodium and chloride traffic in the macula densa and distal convoluted tubule (DCT); elevated sympathetic nervous system activity via recruitment of β_1_-adrenergic receptors; and feedback from humoral factors such as angiotensin I, potassium (renin secretion increases with hypokalemia and decreases with hyperkalemia), and atrial natriuretic peptide (14). Thus, conditions that reduce renal perfusion or sodium delivery to the DCT stimulate the release of renin into the bloodstream (33).

## Dynamics of the intestinal epithelial barrier

### Influence of the intestinal tract on volume composition

Although the cardiovascular and renal systems regulate body fluids, there is evidence of the gastrointestinal tract's role in water and electrolyte balance. Gastric compliance adjusts to extracellular fluid changes, increasing during hypovolemia and decreasing with volume expansion (34).

Acute reduction in blood volume leads to decreased gastric tonus and an increase in gastric compliance compared to baseline values. Nevertheless, intravenous infusion of an isovolemic saline solution normalizes the gastric volume-pressure relationship (35). This phenomenon has been reproduced in both preclinical and clinical studies using a barostat system to monitor gastric tone. In animal models, blood volume replacement during hemorrhagic conditions improves gastric volume responses (36). Additionally, bilateral nephrectomy results in significant alterations in plasma volume, including increased blood pressure, blood volume, potassium levels, and blood osmolarity and accumulation of nitrogenous waste products (37).

The intestinal tract plays a key role in responding to blood volume changes, with studies linking volume shifts to small intestine motility in animal models. Additionally, sympathetic innervation enhances fluid absorption, integrating into the broader water homeostasis system (38).

It has been demonstrated that hypervolemia, as observed in certain pathological conditions such as kidney disease, induces volume-related changes in the colon that result in increased colonic secretion of water and electrolytes (34). This response appears to be progressive, with colonic secretion increasing proportionally to blood volume expansion. The underlying mechanism involves the release of natriuretic peptides, a group of hormones secreted by various tissues, particularly the heart, as previously discussed. These peptides exert pleiotropic effects, with the kidney being the primary target. In the kidneys, they enhance water and sodium excretion by inhibiting the Na^+^/K^+^-ATPase pump and suppressing renin and aldosterone secretion (37). Furthermore, natriuretic peptides induce muscle relaxation in the intestinal wall and blood vessels (39). The intestine's capacity to adapt to acute blood volume fluctuations is physiologically critical. Indeed, in animal models, enterectomy or prolonged fasting significantly impairs blood volume restoration and survival following hemorrhage (40).

### Intestinal morphofunctional barrier: control of hydric and solute fluxes

**Figure 2:**
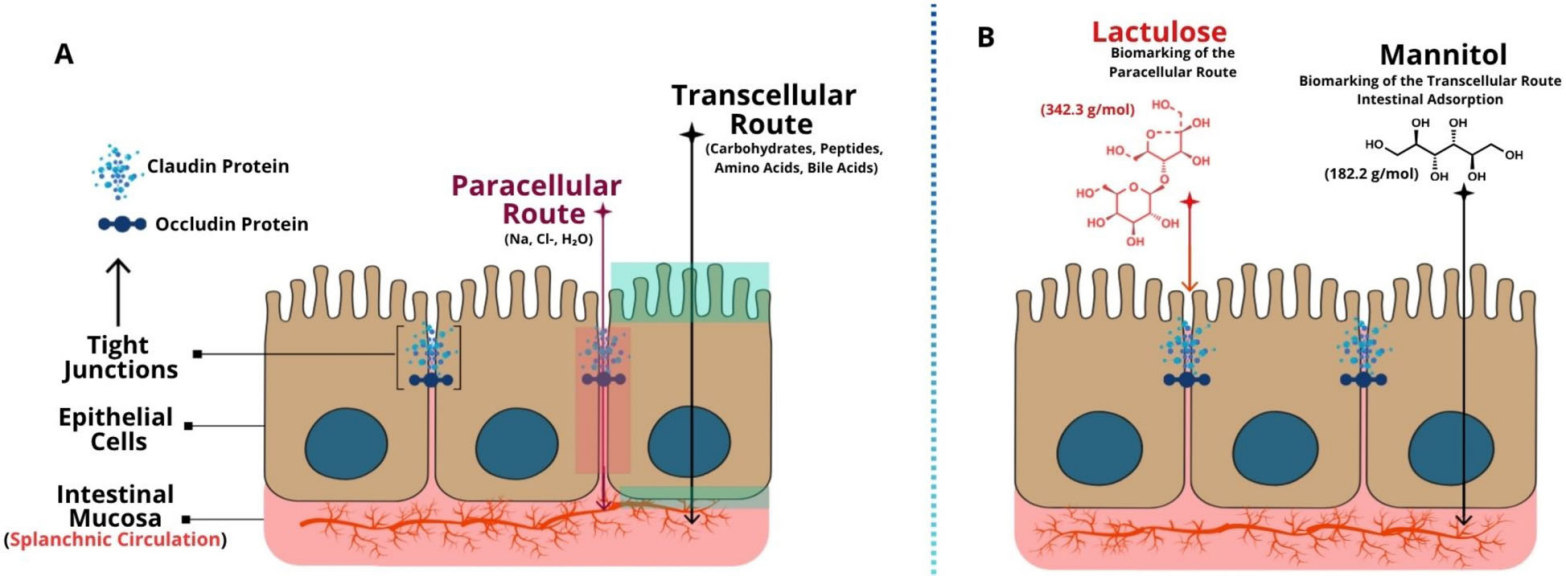
Transcellular transport occurs across cells, involving the passage of substances through the apical and basolateral membranes, while paracellular transport occurs between cells, through the intercellular space. **A**, Sites of transcellular absorption, both apical and basolateral (green) and paracellular route (red). **B**, Chemical structures of the carbohydrates lactulose and mannitol.

The intestinal epithelium, a single-layered cell lining of the intestinal lumen, exerts essential functions in the organism. It is primarily composed of columnar epithelial cells, goblet cells (which produce mucus), enteroendocrine cells (which secrete hormones), and regenerative stem cells (41). This highly polarized cell monolayer forms TJs that restrict access to intercellular spaces, thereby establishing a selective permeability barrier via two main routes: transcellular and paracellular transport (42,43) (see Figure 2). The intestinal barrier function and intestinal permeability are part of a dynamic process that responds to various physiological, pathological, and pharmacological stimuli (43).

The balance between selective paracellular permeability and barrier function is essential for the proper absorption of micronutrients, energy substrates, and water, and for a normal immune defense (5). Under normal physiological conditions, the intestinal epithelium coordinates the interplay between paracellular and transcellular pathways, which are highly interdependent. The main energy substrates are absorbed via various transcellular transporters, notably sodium-glucose linked transporter 1 (SGLT-1) for glucose, glucose transporter 5 (GLUT-5) for fructose, peptide transporter 1 (PepT-1) for peptides, and a group of apical membrane transporters specific for amino acids (44). Complete nutrient absorption requires a second set of transporters located in the basolateral membrane, including glucose transporter 2 (GLUT-2) and proteins specialized in peptide and amino acid transport. The paracellular pathway has a unique complexity and dynamic regulation, allowing permeation of water and small ions such as Na^+^ and Cl^+^ (7). Studies combining electrophysiological and high-resolution imaging techniques have shown that paracellular ion flux - particularly Na^+^ - is essential to maintain ideal intestinal absorption rates. Impairment of this pathway results in malabsorption, hydroelectrolytic imbalance, and increased mortality in *in vivo* models (45). In this scenario, the Na^+^/K^+^-ATPase pump is essential to maintain the electrochemical balance of the enterocyte, also generating the significant function of sodium recycling and its luminal disposition for substrate absorption (5).

Intestinal TJs are the primary regulatory structures for intestinal permeability. This morphofunctional region operates through electrochemical mechanisms governed by key junctional proteins (claudins and occludins) as well as the accessory ZO proteins (46). Based on their electrochemical properties, these proteins determine pore pathway permeability: claudins may either form ion-selective channels or act as sealing components within the TJs complex (6). Channels formed by pore-forming claudins are both charge- and size-selective, allowing passage of solutes with a maximum diameter of approximately 0.6 nanometers (47). The integrity of TJs enables optimal transcellular absorption of micro- and macronutrients via major apical transporters such as SGLT-1 and amino acid-specific carriers (6,44). These processes are essential for preserving cellular fluid composition and volume, as well as ensuring the availability of energy substrates required for overall cellular function (6).

The structural behavior of junctional proteins is considered complex, due to the intricate network of molecular interactions involving not only the proteins themselves but also cytoplasmic elements such as the cellular cytoskeleton (48). These proteins are highly susceptible to modulation by inflammatory markers and by alterations in oxygen and energy supply to intestinal cells (48,49). Although these observations are primarily derived from preclinical models, there is consistent evidence that local inflammation disrupts the functional dynamics of TJs. This phenomenon has been documented in conditions such as malnutrition and energy deficiency and is associated with changes in body composition (6,44,50).

### Junctional proteins orchestrate the paracellular pathway

TJ proteins are situated in the apical region of epithelial enterocytes, where claudins and occludins constitute the central components of the junctional complex, while ZO proteins serve as accessory elements. ZO proteins are crucial for mediating the linkage between transmembrane junctional proteins and the cytoskeletal network of the enterocyte (47). Occludin and claudin exhibit dynamic and self-regulating molecular architectures responsive to physiological stimuli, demanding detailed understanding.

#### Ocludin

Ocludin, discovered in 1993, was the first integral membrane protein identified in epithelial TJs. Its name derives from the Latin *occludere*, meaning ‘to close off' or ‘restrict passage'. Occludin is a 65-kDa membrane-associated tetraspanin phosphoprotein, characterized by four transmembrane domains, two extracellular loops, and one intracellular loop (51). It also features a short N-terminal and a long C-terminal cytoplasmic domain. The homophilic interaction of its extracellular loops with those of neighboring cells contributes to forming a barrier against macromolecules, but not against small ions (52). The extended C-terminal domain interacts with several intracellular TJ proteins, particularly ZO proteins, which are essential for anchoring occludin to the actin cytoskeleton (52).

#### Claudin

Claudins (claudin-1 and -2), identified in 1998, are part of a multigene family, made up of at least 27 members. Its isoforms show a different expression pattern, important for determining the physiological properties of TJs (53). Similar to occludin, claudins are proteins of 20-27 kDa and, morphologically, have one intracellular loop and two extracellular loops, in addition to C-terminal and N-terminal cytoplasmic domains (54). The extracellular loops of claudins engage in both homophilic and heterophilic interactions with claudins on adjacent cells. These interactions contribute to the formation of TJs that function as both a barrier and a selective pore system, regulating the passage of specific molecules through the paracellular route (54). Claudins perform different functions and can be divided into two groups: claudin-1, -3, -4, -5, -8, -9, -11, and -14 can be categorized as barrier-forming claudins, while claudin-2, -7, -12, and -15 are pore-forming claudins (55). Genetic, molecular, and functional studies have shown that the absence of certain claudin subtypes (e.g., claudin-1, -2, -15, and -23) impairs barrier function and triggers a series of complex pathophysiological events. These include growth disturbances, ionic and bioelectrogenic imbalances, macronutrient malabsorption, and morphological alterations in the intestinal epithelium (6,56).

## Physiological intravascular variations affect epithelial cells

### Intestinal epithelial cells respond to variations in osmotic content

There is strong evidence of crosstalk between volemia-dependent mechanisms of the cardiorenal axis and intestinal epithelial cells. Kidney disease disrupts sodium and electrolyte balance, increasing cellular volume, while intestinal epithelial cells, due to their high transport capacity and abundant membrane transporters, are especially vulnerable to these fluctuations (57).

To prevent cellular shrinkage or swelling, enterocytes - like many other cell types - have efficient mechanisms to maintain osmotic balance (58). Volume regulatory mechanisms have been identified in intestinal epithelial cells located in both the villus and crypt regions of the small intestine. Enterocytes have sensors that regulate intracellular fluid composition. Crypt cells that swell in response to decreased extracellular osmotic pressure can restore their original volume via distinct K^+^ and chloride Cl^+^ permeability pathways, which promote the efflux of these ions from the intracellular environment (59). In general, intracellular osmolarity is regulated through the activation of membrane transport pathways that mediate either net solute uptake, referred to as regulatory volume increase, or net solute loss, known as regulatory volume decrease (57,59,60). Regulatory volume increase involves sodium chloride (NaCl) uptake through the activation of sodium/hydrogen (Na^+^/H^+^) and chloride/bicarbonate (Cl^+^/HCO_3_^+^) exchangers, or through sodium-potassium-chloride (Na^+^-K^+^-2Cl^+^) and sodium-chloride (Na^+^-Cl^+^) symporters. In contrast, regulatory volume decrease relies primarily on potassium chloride (KCl) efflux via selective K^+^ and Cl^+^ channels or potassium-chloride (K^+^-Cl^+^) symporters (58).

Intestinal epithelial cells appear to be highly exposed to intravascular changes influenced by mediators of the distribution and composition of blood flow (61). This phenomenon is evident in several conditions, including septic states, while receiving less attention in cases of cardiac rupture and uncontrolled physiological stress caused by physical exercise (62,63). These two conditions are clinically silent but potentially serious and with significant clinical relevance.

### Volemic disruption in heart failure and gut stress

Despite continuous advances over the past decade in understanding the mechanisms underlying the onset and progression of heart failure, mortality and hospital readmission rates remain significantly high. Heart failure has also been associated with intestinal dysfunction (64). Although the mechanisms linking the heart and intestinal systems are uncertain, previous studies have demonstrated a bidirectional relationship, leading to the identification and characterization of the so-called ‘gut-heart axis' (62). The severe effects of heart failure compromise blood distribution and perfusion to organs and tissues, with epithelial cells being particularly affected. Under normal physiological conditions, approximately 25% of cardiac output is directed to the splanchnic circulation, making the intestine one of the most highly perfused organs during periods of rest (65).

In this context, increased sympathetic nervous system activity leads to the constriction of precapillary and postcapillary vessels, reducing blood perfusion. These intestinal vascular changes may occur before noticeable alterations in heart rate or blood pressure, meaning that even slight decreases in cardiac output can result in varying degrees of intestinal ischemia (66).

Protein loss from the gastrointestinal tract is common in patients with right heart failure, particularly in those with congenital heart disease, often presenting as protein-losing enteropathy with symptoms such as hypoproteinemia, edema, and elevated fecal alpha-1-antitrypsin (66). This condition may contribute to cardiac cachexia, along with factors such as anorexia, malabsorption, mental depression, neurohormonal activation, chronic inflammation, and immune abnormalities. Weight loss is independently associated with poorer survival in patients with chronic heart failure (CHF) (66,67).

The increase in intestinal permeability appears to be a critical point in the pathophysiology of heart failure. A clinical study reported an increase in intestinal wall thickness, greater intestinal permeability, and reduced nutrient absorption, indicating intestinal ischemia in patients with CHF (68). Additionally, a greater presence of bacterial biofilms may occur on the intestinal mucosa, contributing to chronic inflammation and malnutrition. These findings underscore the importance of the systemic impact of heart failure, particularly on gastrointestinal function, and its consideration in the clinical management of affected patients.

Disturbances of the intestinal epithelium in the context of heart failure appear to be associated with inflammatory processes. CHF is linked to reduced carrier-mediated intestinal transport - particularly in edematous patients - and to increased levels of bacterial endotoxins, such as lipopolysaccharide (LPS), in the bloodstream, contributing to systemic inflammation (68). Stabilization of heart failure has been shown to reduce circulating LPS levels, suggesting a connection between intestinal dysfunction, ischemia, and endotoxin translocation, and underscoring its role in sustaining chronic inflammation (68).

### Volemic redirection generates gastrointestinal (GI) symptoms during high-intensity exercise

Although no validated biomarker currently links gastrointestinal symptoms to cardiorenal function during intense exercise, critical shifts in cardiac output - reducing perfusion to renal, hepatic, and gastrointestinal systems - are proposed as a key pathophysiological mechanism. At rest, cardiac output is distributed approximately as follows: 20% to skeletal muscle, 22% to kidneys, 27% to the liver, and 7% to other tissues, including the gastrointestinal tract. During strenuous endurance exercise, this distribution shifts dramatically: 84% to skeletal muscle, 1% to kidneys, 2% to liver, and 3% to other tissues, reflecting a substantial splanchnic and renal hypoperfusion (12).

At maximal exercise intensity, gut blood flow can decrease to as little as 20% of its resting value in both trained and untrained individuals, with the sympathetic nervous system activity playing a key role in the redistribution of blood during physical exertion (67). Classical studies by Oktedalen et al. (68) reported that splanchnic blood flow may drop to critically low levels under maximal stimulation, potentially impairing GI motility, intestinal absorption, and mucosal integrity. These changes may contribute to exercise-induced abdominal symptoms and bacterial translocation.

High-intensity physical exercise increases intestinal permeability due to oxidative stress. A recent clinical model demonstrated that prolonged intense exercise causes damage to TJ proteins, such as claudin-1, and increases oxidative protein levels in intestinal tissue, leading to sustained intestinal barrier dysfunction even at rest (69). In an experimental model, our group reported a significant impact on the paracellular pathway in rodents subjected to exhaustive swimming training, with increased intestinal permeability and altered claudin-2 gene expression (70).

A clinical trial with athletes found that high-intensity interval running significantly increases markers of GI damage, including elevated intestinal permeability and intestinal fatty acid-binding protein (I-FABP) levels. Interestingly, these changes occurred without a clear correlation with reported GI symptoms, which remained mild or absent (71). These findings suggest that although acute high-intensity exercise can compromise intestinal integrity, it does not necessarily lead to overt clinical discomfort. On the other hand, other studies have reported substantial endotoxemia in athletes. Jeukendrup et al. (72) observed that 93% of triathletes experienced GI symptoms during or after a triathlon, with 45% reporting severe complaints. Mild endotoxemia, characterized by elevated circulating LPS levels, was detected in 68% of participants after a race. Additionally, they present a 27-fold increase in interleukin-6 (IL-6) and a 20-fold rise in C-reactive protein (CRP), indicating a pronounced acute-phase inflammatory response. A previous study found that during acid-base disturbance triggered by high-intensity exercise in a preclinical model, the use of sodium bicarbonate showed protective action against intestinal changes, indicating future approaches for intervention (73).

Similar patterns were noted in a 24-h continuous ultramarathon conducted under temperate conditions, resulting in circulatory endotoxemia and pro-inflammatory cytokinemia, counterbalanced by a compensatory anti-inflammatory response (74). These data underscore the complexity of intestinal responses to strenuous physical exertion. Therefore, detecting subtle variations in intestinal epithelial function remains a significant challenge, requiring the use of sensitive analytical techniques and specific biomarkers.

### Analytical techniques and clinical biomarkers of intestinal permeability

Assessing pathophysiological variations in the intestinal epithelium presents important challenges due to its structural complexity, dynamic adaptive responses, and the need for specific and sensitive methods to detect subtle changes. In both clinical and experimental settings, the main investigative approaches include: endoscopy with biopsy, which enables direct visualization of the gastrointestinal tract and collection of tissue samples for histological analysis; immunohistochemistry, which uses specific markers to identify cellular and molecular alterations in the intestinal epithelium; non-invasive fecal tests, which assess inflammatory markers, protein loss, and intestinal permeability; and chromoendoscopy, which employs dyes to highlight suspicious areas during endoscopic examination (75,76).

These methods are commonly applied in the diagnosis of intestinal diseases such as Crohn's disease, ulcerative colitis, irritable bowel syndrome (IBS), and environmental enteropathy, in order to identify events related to intestinal infections and inflammatory processes (76). In this context, a set of biomarkers used to evaluate pathobiological processes involving the morphofunctional intestinal barrier is presented in Supplementary Table S1, including immunological markers, indicators of inflammatory status, and metabolic parameters (41).

Furthermore, few studies have employed these useful techniques to investigate the effects of intravascular volume variations in the context of heart failure and physiological stress induced by high-intensity physical exercise. This limitation may be attributed to several factors, including high costs, technical complexity, lengthy processing times, limited availability of resources, patient-related constraints, and suboptimal diagnostic accuracy.

However, the use of oral carbohydrate-based tests has proven to be effective in assessing the intestinal morphofunctional barrier. Glycids commonly used in these tests include lactulose, mannitol, sucrose, xylose, and rhamnose. Among them, the lactulose-mannitol test is the most widely employed. Lactulose, a disaccharide, is absorbed by enterocytes via the paracellular pathway, whereas mannitol, a monosaccharide, is absorbed through transcellular transport across cell membranes (77) (Figure 2B).

The analytical method involving these two saccharides consists of their simultaneous oral ingestion, diluted in water, followed by urine collection over a period of 2 to 5 h. Quantification is reliably performed using high-performance liquid chromatography (HPLC) (78). Under normal epithelial conditions, an increase in urinary lactulose excretion reflects enhanced intestinal permeability, indicating functional and structural impairment of the paracellular route. Changes in mannitol excretion, either increased or decreased, reflect alterations in the epithelial absorptive surface area.

The lactulose-to-mannitol excretion ratio (L:M) is widely used to assess overall intestinal epithelial permeability. Alterations in this ratio have been associated with several intestinal disorders involving immunological, inflammatory, or environmental etiologies (10,79,80). This approach has also proven effective in models of metabolic dysfunction, where epithelial changes may be subclinical or asymptomatic (81).

Our research group has been documenting pathobiological variations in the intestinal morphofunctional barrier through the lactulose-mannitol (LM) test, applying it in both *in vivo* and clinical trials (49). For decades, this technique has proven effective in detecting increased intestinal permeability in the context of environmental enteropathy, malnutrition, and infectious diseases, as well as in preclinical models of diabetes, malnutrition, and the physiological impact of exhaustive physical exercise (52,70,82,83).

Although the LM test is efficient for evaluating intestinal permeability, the highly dynamic nature of the intestinal epithelium underscores the need for more robust analytical methodologies. In this context, there is growing interest in the use of a coupled liquid chromatography and the tandem mass spectrometry platform (LC-MS/MS), offering greater analytical precision and improved ergonomic performance in detecting LM-derived biomarkers (84).

### LC-MS/MS for intestinal permeability assessment in heart failure and physical exercise: a promising approach yet to be explored

It is well established that the LM test reflects both pathophysiological and morphofunctional aspects of the intestinal epithelium. Therefore, optimizing analytical efficiency in this context has become increasingly important. In light of recent laboratory advances, it is recommended that conventional HPLC methods be upgraded to LC-MS/MS techniques. This platform offers improved precision, specificity, and sensitivity for detecting LM-derived biomarkers.

Kubica et al. highlighted the high accuracy of LC-MS/MS in quantifying urinary lactulose and mannitol as a method to detect altered intestinal permeability in children with inflammatory bowel diseases, including chronic intestinal inflammation, gastric ulcers, and duodenal ulcers (85). Similarly, the method has shown high specificity and sensitivity in identifying LM markers in pediatric patients with irritable bowel syndrome (86). Our research group has recently used the LM test employing the LC-MS/MS platform and found increased paracellular intestinal permeability in children from low-income backgrounds, thereby providing further support for its applicability in clinical and epidemiological research (87).

Studies investigating intestinal permeability in patients with heart failure using advanced analytical techniques remain scarce. However, a previous approach employing liquid chromatography identified increased intestinal epithelial permeation of melibiose and rhamnose, along with altered carrier-mediated absorption - both active (3-O-methyl-D-glucose, 3-OMG) and passive (D-xylose) - which were associated with elevated inflammatory markers (68). In this context, Sandek et al. (67) reported significant morphological and functional enteric alterations in patients with CHF, including reduced intestinal perfusion, mucosal edema, increased intestinal permeability, impaired immunological defense, and enhanced bacterial biofilm formation. Thus, these studies underscore the need to refine more specific and sensitive methodologies to assess intestinal permeability in this population.

To date, in the context of physical exercise, there is still limited information on variations in intestinal permeability using LC-MS/MS. However, a recent controlled clinical study investigated intestinal permeability using lactulose and rhamnose as biomarkers through this analytical approach and found no significant changes in gastrointestinal symptoms previously associated with collagen supplementation (88).

**Figure 3:**
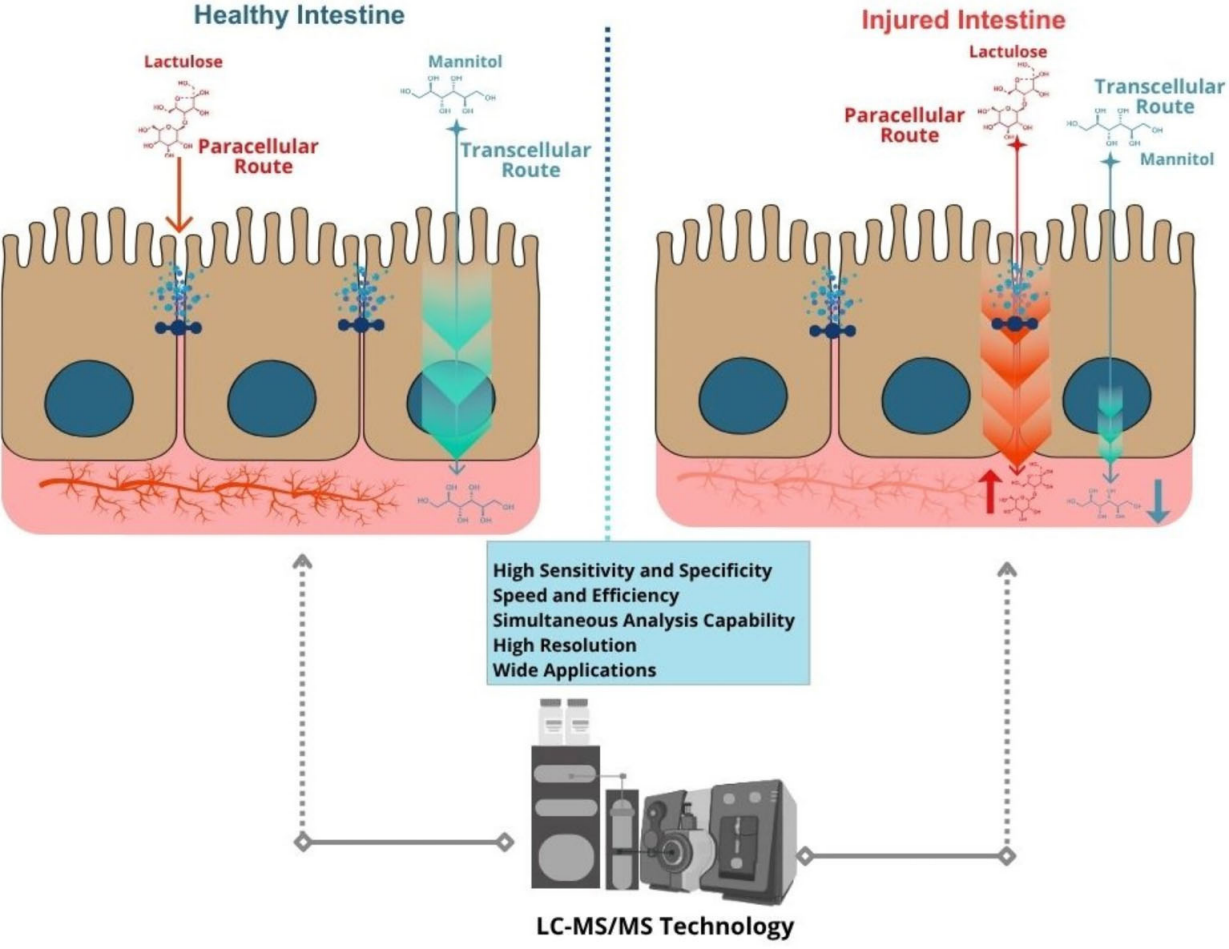
Use of coupled liquid chromatography and tandem mass spectrometry (LC-MS/MS) platforms to improve the identification of intestinal epithelial damage in pathophysiological conditions that reduce perfusion and oxygen supply to the epithelium.

While HPLC remains widely used for its simplicity and cost-effectiveness, LC-MS/MS offers superior sensitivity, specificity, and multiplexing (89). However, its broader application is limited by its high costs and the need for specialized personnel. In contexts such as cardiac failure, especially in critical care, and exercise-induced intestinal injury, precise analytical methods like LC-MS/MS are essential for accurate real-time assessment. A recent review highlights the clinical relevance and diagnostic potential of these advanced techniques for both contexts (89). In clinical intervention, this approach can accurately present mechanisms of transient damage to the intestinal epithelium (90). A growing body of evidence supports the application of intestinal biomarkers assessed by advanced analytical techniques, particularly in conditions that significantly impact the integrity of the intestinal epithelium. In addition to the lactulose and mannitol biomarkers, Supplementary Table S2 presents immunoinflammatory markers applied in the diagnosis of intestinal disorders that could be validated in mass spectrum approaches to increase the identification range (41,90). The use of LC-MS/MS appears especially promising in contexts involving intravascular volume fluctuations and altered tissue perfusion, such as in heart failure and high-intensity physiological stress (Figure 3).

A strategic approach is essential to overcome diagnostic limitations and enable a more accurate, integrative assessment of the cardiorenal volume-intestinal axis. This underscores the need for a validated diagnostic framework, in which a reliable evaluation of intestinal permeability using lactulose and mannitol depends on rigorous protocol standardization, control of confounding factors, and robust LC-MS/MS validation. Furthermore, integrating these elements with correlations of hemodynamic and inflammatory biomarkers - particularly those listed in Supplementary Tables S1 and S2 of this review - and including appropriate control groups ensures not only clinical relevance and reproducibility, but also meaningful translational impact.

## Conclusions

Intravascular volume regulation relies on a complex network of physiological mediators that respond to homeostatic fluctuations under both physiological and pathophysiological conditions. Among these mechanisms, the renal-cardiac axis plays a central role in adapting to volume changes. The intestinal epithelium is highly sensitive to both minimal and critical variations in the flow and composition of intra- and extravascular fluids, making it one of the systems most affected by organ hypoperfusion during heart failure and high-intensity physical exercise.

In this context, the LM test has proven effective in detecting alterations in the absorptive surface area and functional changes related to paracellular transport, thereby indirectly reflecting TJ integrity. It also minimizes the risks associated with invasive procedures while enhancing sensitivity in detecting subtle epithelial disruptions.

Finally, updating the analytical method used to quantify these biomarkers through LC-MS/MS is highly recommended. This technique offers superior performance in terms of speed, sensitivity, and accuracy, and represents a promising tool for investigating intestinal permeability in conditions of low cardiac output and in individuals undergoing high-intensity physical exertion, such as endurance athletes.

## Supplementary Materials

Supplementary Material Click to view [pdf].

## Data Availability

All data generated or analyzed during this study are included in this published article.
