# Supplementary material for: Interplay between volemic balance and the intestinal tract: insights on biomarkers and diagnostic tests used to assess intestinal morphofunctional barrier
Source: Braz J Med Biol Res. 2026 Jan 30;59:e15041. doi: 10.1590/1414-431X2025e15041 (PMC12858124; doi:10.1590/1414-431X2025e15041)
Supplement: Supplementary Material [file 1414-431X-bjmbr-59-e15041-suppl.pdf]

Table S1. Main mediators of the cardiorenal, nervous, and endocrine systems.

**Regulation of body fluids under physiological conditions**					
**Cardiac**		**Renal**		**Endothelial**	
**Preload – contractility – postload**.		**Regulate osmolality, electrolyte balance and acid-base balance**		**Control of vascular tone**	
**Mediator**	**Actions**	**Mediator**	**Actions**	**Mediator**	**Actions**
**Atrial Natriuretic Peptide (ANP)**	↓ Regulates intravascular volume through the relaxing effect it produces on vascular smooth muscle cells;<br>↓ Blood pressure by reducing sympathetic tone and inhibiting endothelin-1 (ET-1)/secretion and decreases sodium retention;<br>↑ Antagonist of RAAS.	**Prostaglandins (PGE2 and PGD2)**	↑ Vasodilation	**Nitric Oxide (NO)**	↑ Relaxation of arterial and venous vasculature
		**Thromboxane A (TXA)**	↑ Vasoconstriction	**Prostacyclins**	↑ Vasodilation
		**Atrial Natriuretic Factor (ATF)**	↑ Excretion of sodium and water by the kidneys	**Thromboxane A (TXA)**	↑ Vasoconstriction
		**Nitric Oxide (NO)**	↑ Relaxation of the venous and arterial vasculature.	**Angiotensin II**	↑ Vasoconstriction
		**Prostacyclin (PGI2)**	↓ Extracellular volume stimulates.		
		**Endothelium-derived Hyperpolarizing Factor (EDGF)**			
		**Renin**	↑ Angiotensin I;<br>↑ Angiotensin-converting enzyme activity;<br>↑ Angiotensin II;<br>↑ Aldosterone;<br>↑ Aldosterone release;<br>↑ Reabsorption of sodium and water by the kidneys;<br>↑ Constriction of blood vessels, increasing blood pressure.	**Endothelin-1**	↑ Vasoconstrictor and modulates vasomotor tone.
				**ANP**	↑ RAAS antagonist.
				**Reactive Oxygen Species**	↑ Vasodilator response.
**Sympathetic Nervous System**		**Inflammatory Signaling**		**Parasympathetic Nervous System**	
**Catecholamines (Noradrenaline/ adrenaline)**	↑ Blood pressure and peripheral vascular resistance.<br>↑ Positive inotropic and chronotropic effects,<br>↑ Redistribution of peripheral blood volume for maintenance of organic perfusion;<br>↑ Activation of the RAAS system.	**Interleukin-1 (IL-1)**<br>**Interleukin-6 (IL-6)**<br>**Tumor Necrosis Factor (TNF-alpha)**	↑ Inflammatory status, endothelial dysfunction.<br>↑Intravascular coagulation.<br>↑ Uncoupling of beta-adrenergic stimulus and generation of free radicals.	**Acetylcholine**	↑ Vasodilation and reduction of heart rate from the decrease in contraction force and cellular metabolism.
**Enteric Signaling**					
Activation of guanylate cyclase C receptors					
	**Mediators**			**Actions**	
	**Guanylin and uroguanylin**			Promotes natriuresis.<br>Regulates sodium balance.<br>↑ Secretion of water and electrolytes such as sodium, chloride and potassium.<br>↑ Urinary flow.<br>Synergistic effect with ANP.	

Table S2. Summary of the main biomarkers used in the clinical setting for the analysis of intestinal permeability in the functional, inflammatory, and immunological context. Adapted from Rodrigues and collaborators 2016 (41; doi: 10.5151/9788580391893-18). HPLC: high-performance liquid chromatography; LC-MS/MS: coupled liquid chromatography and the tandem mass spectrometry platform; ELISA: enzyme-linked immunosorbent assay; IIF/ELISA: Indirect immunofluorescence.

	Biomarkers	Sample	Method	Function	Pathobiological Biomarking
**Electrophysiological parameters of intestinal epithelial cells**	Specific substrate (glucose, peptide amino acid) marked substance (Cr-EDTA)	Intestinal tissues (duodenum, jejunum, ileum, and colon)	Using Chambers	Classic method, widely used to verify in intestinal tissues the bioelectrogenic parameters involved in the absorption of nutrients and permeation, generally associated with the damage processes in epithelial cells.	Various intestinal diseases associated with inflammation and malnutrition among others.
**Absorption, permeability, damage and intestinal repair**	% Excretion of lactulose (%L; 342 k Da)	Urine	HPL and/or LC-MS/MS platform	The %L assesses the rise in permeability or the damage to the intestinal epithelium.	%L is linked to diseases that modify the permeability and/or cause damage to the functional gastrointestinal tract barrier.
	% Excretion of mannitol (%M; 182 kDa)	Urine	HPL and/or LC-MS/MS platform	%M evaluates the area of intestinal absorption.	%M is linked to diseases that alter the area of intestinal absorption.
	Lactulose rate/mannitol (L:M)	Urine	HPL and LC-MS/MS platform	The L:M ratio is used to evaluate the area absorption, injury and functional intestinal barrier repair.	The LM ratio has proven to be a sensitive test for assessing changes in permeability, absorption, and damage to the functional intestinal barrier.
	Ileal Fatty Acids Binding Protein (I-FABP; ~13-14 kDa)	Plasma, serum, urine	ELISA	Located in the epithelial cells of the small intestine, it plays a crucial role in the utilization, transport, and metabolism of fatty acids.	This is a biochemical marker indicating that the intestinal cell has been chemically damaged, as evidenced by low levels or absence of I-FABP.
	Diamine oxidase (DAO; ~91 kDa)	Serum	ELISA	DAO is produced in the intestinal mucosa and has an inverse relationship with intestinal permeability.	DAO is associated with the integrity of the membrane and the maturity of cells within the small intestinal mucosa.
	Litostatine-1-Beta (Reg1β; 19 kDa)	Fecal	ELISA	This protein is synthesized in the crypt cells of the intestine and is involved in tissue repair, cell growth, and proliferation.	The presence of this protein in stool samples indicates the proliferation of crypt cells.
**Intestinal Inflammation**	Marked Leukocytes	Fecal	Indium-111-labeled white blood cell scintigraphy	Radiolabeled leukocytes are regarded as the gold standard for detecting inflammation in fecal samples.	This suggests a rise in inflammatory mediators due to increased intestinal permeability.
	Alpha-1-Antitrypsin (α-1-AT; 52 kDa)	Fecal, serum	ELISA	α-1-AT is a glycoprotein, acute phase protein and proteinase inhibitor.	Its presence in the fecal sample reflects an increase in intestinal permeability during the inflammatory process.
	Alpha 2-macroglobulin (α2M, 820 kDa)	Fecal, Serum	ELISA	This glycoprotein functions as an antiproteinase, with the ability to inactivate a broad range of proteinases.	It regulates inflammatory responses and inhibits the proteolytic activity of trypsin, plasmin, and kallikrein. It is associated with Crohn's disease but not with ulcerative disease.
	Lactoferrin (LAF; 80 kDa)	Fecal	ELISA	LAF is found in neutrophil granules and exhibits antimicrobial properties.	A high concentration indicates intestinal inflammation.
	Calprotectina (CAP; 36.5 kDa)	Fecal, plasma, serum, urine	ELISA	CAP is a protein that binds calcium and zinc and is part of the S100 protein family. It is produced in neutrophil granulocytes and is also referred to as MRP-8/14, calgranulin A/B, or S100A8/A9.	CAP is present in the cytoplasm of neutrophils, macrophages and eosinophils of ileal tissue. It has antimicrobial, immunomodulatory and antiproliferative effects. It is a potent neutrophil chemotactic factor.
	Myeloperoxidase (MPO; 150 kDa)	Fecal, urine	ELISA	MPO is found in neutrophil granules and facilitates the oxidation of substances using $H2O_2$.	The MPO-$H2O_2$ system exerts a toxic effect on microorganisms and serves as an indicator of inflammatory activity within the intestine.
	Neopterin (NEP; 253 kDa)	Fecal, plasma, serum, urine	ELISA	NEP is produced and secreted by macrophages and dendritic cells as a result of the cellular immune response to stimulation with IFN-g.	NEP is linked to the activation of the cellular immune response.
	Neutrophilic elastase (NE; 29 kDa)	Fecal	ELISA	It is a serine protease found in the primary granules of polymorphonuclear leukocytes, which are released when neutrophils are activated.	Neutrophilic elastase can dissolve the extracellular matrix and serves as a marker for inflammatory diseases.
**Systemic inflammation and immune response**	Anti-neutrophil cytoplasm (ANCA;150 kDa)	Plasma	IIF/ELISA	These antibodies target neutrophil granules and are associated with patients who have ulcerative disease, but not Crohn's disease.	Suggests chronic inflammation linked to heightened intestinal permeability.
	Anti-saccharomyces antibodies (ASCA)	Plasma	ELISA	Similar to the cell wall of enterobacteria, it is associated with patients who have Crohn's	Suggests chronic inflammation linked to heightened intestinal permeability.

				disease, but not with ulcerative disease.	
	Liposaccharide (LPS; ~20 KDa)	Plasma, Serum, Homogenized tissue	ELISA	LPS is a toxin produced by bacteria and released into the environment.	Elevated levels of LPS can trigger inflammatory and anticoagulant effects.
	LPS-Binding Protein (LBP; 51 kDa)	Plasma, Serum	ELISA	LBP is an acute-phase protein continuously produced by the liver. It facilitates the monomerization of LPS and its subsequent transfer to sCD14 and lipoproteins.	This protein is involved in both activating monocytes through LPS and neutralizing LPS via the lipoprotein pathway.
